# A Resonant Pressure Sensor Based upon Electrostatically Comb Driven and Piezoresistively Sensed Lateral Resonators

**DOI:** 10.3390/mi10070460

**Published:** 2019-07-08

**Authors:** Xiaoqing Shi, Sen Zhang, Deyong Chen, Junbo Wang, Jian Chen, Bo Xie, Yulan Lu, Yadong Li

**Affiliations:** 1 State Key Laboratory of Transducer Technology, Institute of Electronics, Chinese Academy of Sciences, Beijing 100190, China; 2 University of Chinese Academy of Sciences, Beijing 100049, China

**Keywords:** resonant pressure microsensor, electrostatic excitation, piezoresistive detection, double-ended tuning forks, comb drive, MEMS

## Abstract

This study proposes a microfabricated resonant pressure sensor in which a pair of double-ended tuning forks were utilized as resonators where comb electrodes and single-crystal silicon-based piezoresistors were used for electrostatic excitation and piezoresistive detection, respectively. In operations, pressures under measurements deform the pressure-sensitive diaphragm to cause stress variations of two resonators distributed on the central and side positions of the pressure-sensitive diaphragm, where the corresponding changes of the intrinsic resonant frequencies are then captured piezoresistively. The developed resonant pressure sensors were fabricated based on MEMS with open-loop and closed-loop characterizations conducted. Key sensing parameters including quality factors, differential pressure/temperature sensitivities and fitting errors were quantified as higher than 17,000, 48.24 Hz/kPa, 0.15 Hz/°C and better than 0.01% F.S. (140 kpa), respectively. In comparison to previously reported resonant pressure sensors driven by parallel-plate electrodes, the developed sensor in this study is featured with a lower temperature sensitivity and a higher stability.

## 1. Introduction

Currently, resonant pressure sensors utilizing mechanical resonators as stress gauges are commonly used in the areas of meteorology and aviation [1] because of significant advantages including large resolutions, high compatibilities with digital instruments, and long-duration stabilities [2]. Mechanical resonators can be classified based on the mechanisms of excitation and detection (e.g., electrothermal excitation and piezoresistive detection [3,4], electromagnetic excitation and electromagnetic induction detection [5,6,7], electrostatic excitation and piezoresistive detection [8,9], electrostatic excitation and capacitive detection [10,11,12]). Among different excitation and detection mechanisms, electrostatic excitation and piezoresistive detection rely on electrostatic forces to drive the resonators into vibration and stress variations of the piezoresistors in the resonators for detection of vibration. In comparison to other modes of excitation and detection, this mechanism is characterized by low energy consumption (i.e., less currents in electrostatic excitation), small device volume (i.e., no requirement of magnets), low detection impedance and low noise levels (i.e., low impedance values of piezoresistors).

Leveraging these advantages of electrostatic excitation and piezoresistive detection, previously, we developed a double-ended tuning fork based resonant pressure sensor [13] featured with an accuracy better than 0.01% F.S. (140kpa) However, parallel-plate capacitor drive electrodes were used in the previously reported resonant pressure sensor, which suffered from limitations of negative stiffness, leading to a drive-bias-induced frequency shift and further degradation of the closed-loop frequency stabilities.

The work described in this paper is a development of the previous study where the resonator was excited by comb-drive capacitor electrodes rather than parallel-plate capacitor drive electrodes. The following sections describe the design and analysis, fabrication, results and discussion for the newly proposed resonant pressure sensor.

## 2. Design and Simulations

**Figure 1:**
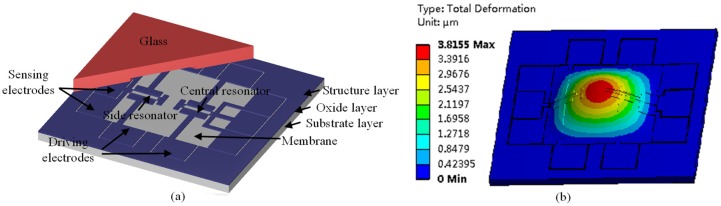
(**a**) Schematic of the microfabricated resonant pressure sensor in which a pair of double-ended tuning forks were utilized as resonators where comb electrodes were used for electrostatic excitation and single crystal silicon based resistors were used for piezoresistive detection. (**b**) Pressure under measurements causes the deformation of the pressure-sensitive diaphragm, leading to frequency shift of the resonators.

The resonant pressure sensor comprised a substrate layer, an oxide layer, a structure layer and a glass cover (see Figure 1a). The substrate layer consisted of a pressure-sensitive diaphragm and electrode vias. The oxide layer was used for electrical isolation between the substrate layer and the structure layer. The structure layer comprised two 40 μm thick resonators suspended on the pressure-sensitive diaphragm and ten electrodes including four driving electrodes and six sensing electrodes. One resonator was on the central position of the pressure-sensitive diaphragm while the other was on the side, enabling differential outputs. The glass cover was used to form a vacuum chamber to protect the resonators which were released. The resonators based on double-ended tuning forks were excited electrostatically into a resonant mode by comb-drive capacitor electrodes. The vibration of the resonators was detected via a pair of single crystal silicon based piezoresistors on the end of the resonant beams.

Figure 1b shows the working principle of the proposed resonant pressure sensor. Upon the application of a pressure, the diaphragm deformed, so stressing the resonators mounted on the diaphragm and modulating their resonant frequencies, which were further detected by piezoresistive resistors. More specifically, the stress was positive in the central region, and thus the central resonator was under positive stress with its corresponding intrinsic resonant frequency increased. Meanwhile, the stress was negative in the side region, and thus the side resonator was under negative stress with its corresponding intrinsic resonant frequency decreased. In addition, in this study, comb-drive capacitor electrodes rather than parallel-plate capacitor drive electrodes were adopted to drive the resonators, significantly improving the performance of the resonant pressure sensor.

The aim of this study was to design a stress-sensitive and temperature-insensitive resonator which vibrated in a lateral dynamically balanced mode. The resonator had an intrinsic frequency of about 100 kHz with a frequency shift of 10% at a full-scale pressure range, which could produce a linear relationship between the applied pressure and the resonant frequency of the resonator based on previously reported experiments [8].

**Figure 2:**
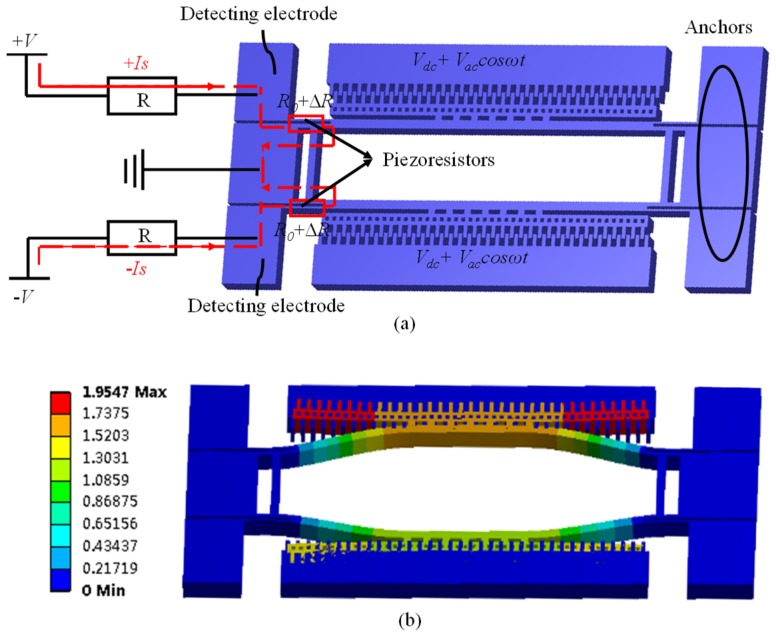
Setup (**a**) and simulation (**b**) of double-ended tuning forks relying on comb drive actuation and piezoresistive detection.

The resonator in this study was characterized by double-ended tuning forks excited by comb-drive electrodes as shown in Figure 2a. The moving comb fingers were not directly connected with the resonant beams, guaranteeing the same displacements for each finger in resonant modes when resonant beams bent (see Figure 2b). Some holes were designed in comb fingers to ensure resonant beams were released completely and could vibrate when driven. The piezoresistors lay at the end of the resonant beams with maximum stresses in resonant modes, enabling the quantification of resonant frequency.

The advantages of the comb-drive capacitor electrodes are that they can generate linear excitation forces, which can eliminate the effect of negative stiffness for the resonant beams and can address the potential concern of pull-in effects compared to parallel-plate capacitor drive electrodes. Furthermore, the temperature sensitivity of the resonator excited by comb-drive capacitor electrodes is less than the resonator excited by parallel-plate capacitor drive electrodes, which can increase the stability of the sensor.

Figure 2a shows the electric connection diagram of the resonator which was excited into laterally out-of-phase vibration mode by applying bias voltage signals of the form *V_dc_ + V_ac_cosωt* to two comb-drive capacitor electrodes simultaneously, reducing the energy loss caused by coupling with its supports and improving the quality factor of the resonator [14].

Each resonator possessed two piezoresistors whose resistance changed along with the vibration of the resonant beams. Suppose the initial resistance of piezoresistor is *R*_0_ when there is no stress acting on the piezoresistors. According to the piezoresistive effect of single crystal silicon, the resistances of piezoresistors drift (Δ*R*) when stresses change. When applying opposite constant current in two piezoresistors, the vibration frequency of the resonant beams can be detected by obtaining the differential voltage of the detecting electrodes, which can eliminate common-mode interference and improve the accuracy of the sensor.

## 3. Fabrication

**Figure 3:**
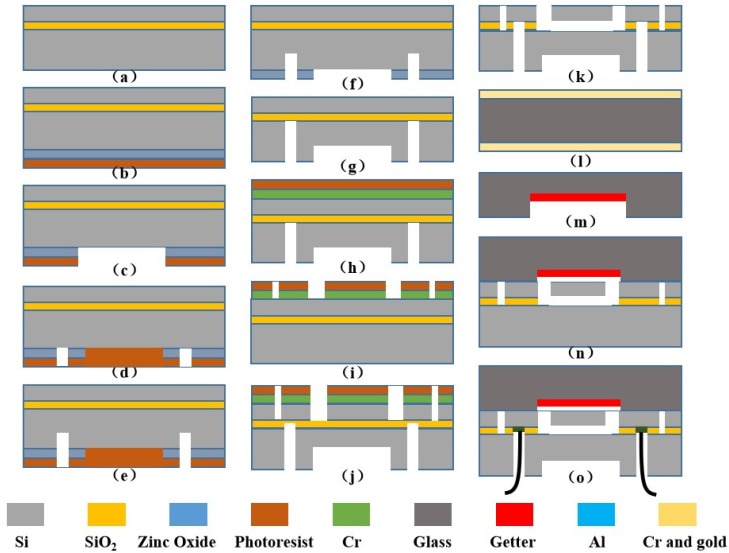
Fabrication process of the resonant pressure sensor: (**a**) thoroughly clean the SOI wafer; (**b**) sputter zinc oxide and spin coat photoresist on the back side of SOI wafer; (**c**,**d**) pattern the pressure-sensitive diaphragm and through-silicon vias; (**e**) etch the through-silicon vias to a certain depth; (**f**,**g**) etch the pressure-sensitive diaphragm and through-silicon vias simultaneously; (**h**) sputter Cr and spin coat photoresist as masks on the front side of SOI wafer; (**i**,**j**) pattern and fabricate the structure layer including resonators, excitation and detection electrodes; (**k**) release the resonators and remove the oxide on top of through-silicon vias; (**l**) sputter Cr and Au materials on both sides of glass; (**m**) process the glass cover with grooves followed by the deposition of getter materials using a hard mask after removing the Cr and Au materials; (**n**) conduct silicon-to-glass anodic bonding; (**o**) sputter Aluminum and form electrical connection.

MEMS based microfabrication (e.g., lithography, etching and bonding) was utilized to form the resonant pressure sensors where a 4-inch SOI wafer with key parameters of a 300 μm substrate layer, a 2 μm oxide layer, and a 40 μm structure layer ((100) plane and <110> crystal orientation) was used. First, the SOI wafer was cleaned by concentrated sulfuric acid and hydrogen peroxide (see Figure 3a). Next, zinc oxide was deposited and patterned using photoresist on the substrate layer to define the pressure-sensitive diaphragm (see Figure 3b,c). The silicon-through vias were etched to a certain depth by deep reactive ion etching (DRIE) using patterned photoresist as the mask (see Figure 3d,e). The photoresist was removed to form both the pressure-sensitive diaphragm and silicon-through vias using patterned zinc oxide as the etch mask (see Figure 3f,g). Resonators were fabricated on the structure layer based on DRIE using patterned Cr and photoresist as the etch mask (see Figure 3h–j). The underlying oxide layer was removed to release the resonators and expose through-silicon vias using gaseous hydrofluoric acid (see Figure 3k). Then, patterned Cr and Au were used as masks to form grooves on a glass wafer, followed by the deposition of getter materials using a hard mask after removing Cr and Au materials (see Figure 3l,m). Anodic bonding was conducted between the patterned SOI and glass wafers to form a vacuum chamber (see Figure 3n), effectively isolating resonators from the outside to guarantee the quality factor of the sensor with the help of deposited getter materials that absorb gases released by the glass wafer in anodic bonding. In the end, Al was sputtered on the backside of the bonded wafer as the pads to form electrical connections (see Figure 3o).

**Figure 4:**
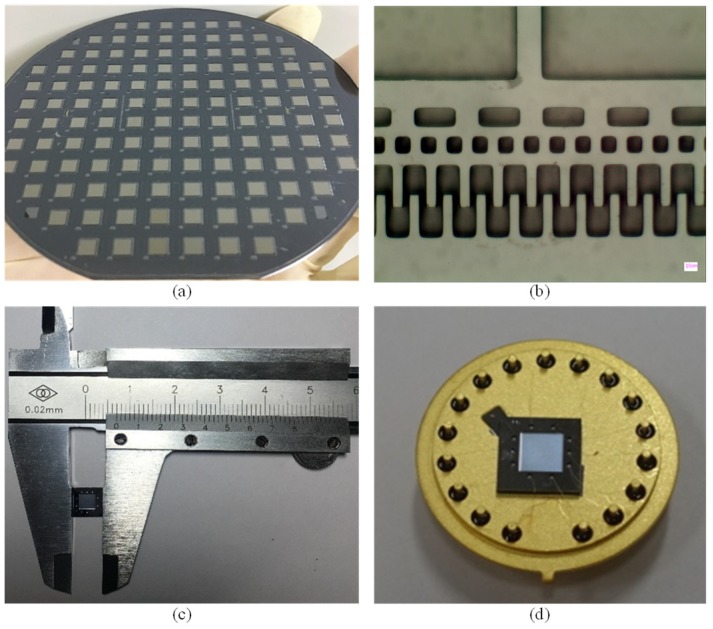
(**a**) Photograph of a patterned wafer after anodic bonding between the SOI wafer and the glass cover with gloves where getter materials was sputtered. (**b**) Microscopic image of detailed comb-drive electrodes in the fabricated resonator. (**c**) Photograph of a prototype sensor with a dimension of 7 mm × 7 mm. (**d**) Photograph of a fabricated sensor mounted on a covar header.

Figure 4a shows the image of a patterned wafer after anodic bonding. Figure 4b shows the microscopic image of detailed comb-drive electrodes in the fabricated resonator. Figure 4c shows the image of a prototyping sensor with a dimension of 7 mm × 7 mm. Figure 4d shows the image of a fabricated sensor with electrical connection with surrounding circuits.

## 4. Results and Discussion

**Figure 5:**
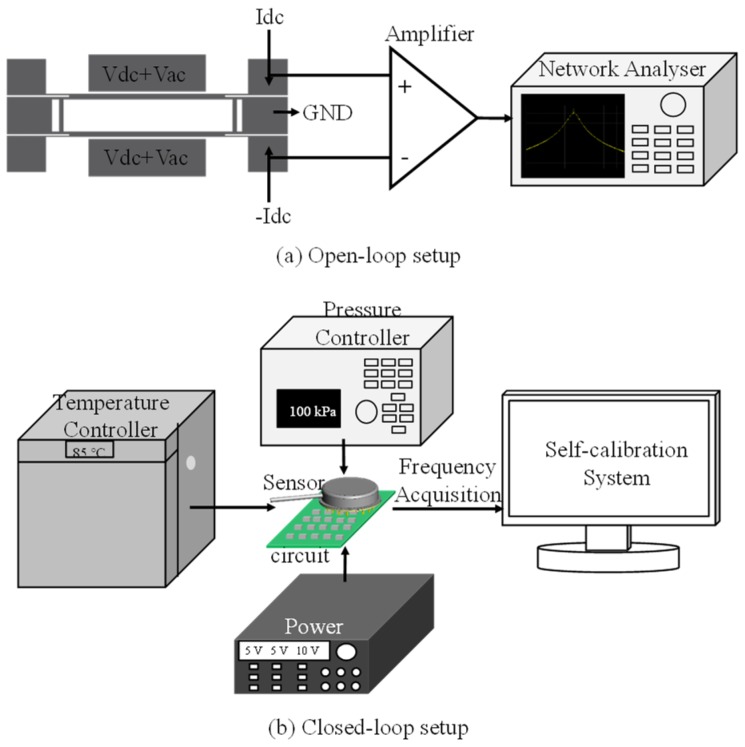
Schematic of open-loop (**a**) and closed-loop (**b**) platforms to characterize the developed resonant pressure sensors with variations of pressure and temperature parameters well under control.

A number of fabricated resonant pressure sensors were characterized by open-loop and closed-loop test systems. In open-loop testing, an E5601B Network Analyzer (Agilent, Santa Clara, CA, USA) was used to obtain the amplitude ratio between the output of the sensor to the input generated by the network analyzer as a function of frequencies (see Figure 5a). In closed-loop testing, the testing parameters including pressure and temperature values were fine-tuned by a pressure controller and a temperature controller, respectively, enabling the collection of the relationship between pressure and temperature under measurement with the intrinsic frequencies of the resonators (see Figure 5b).

**Figure 6:**
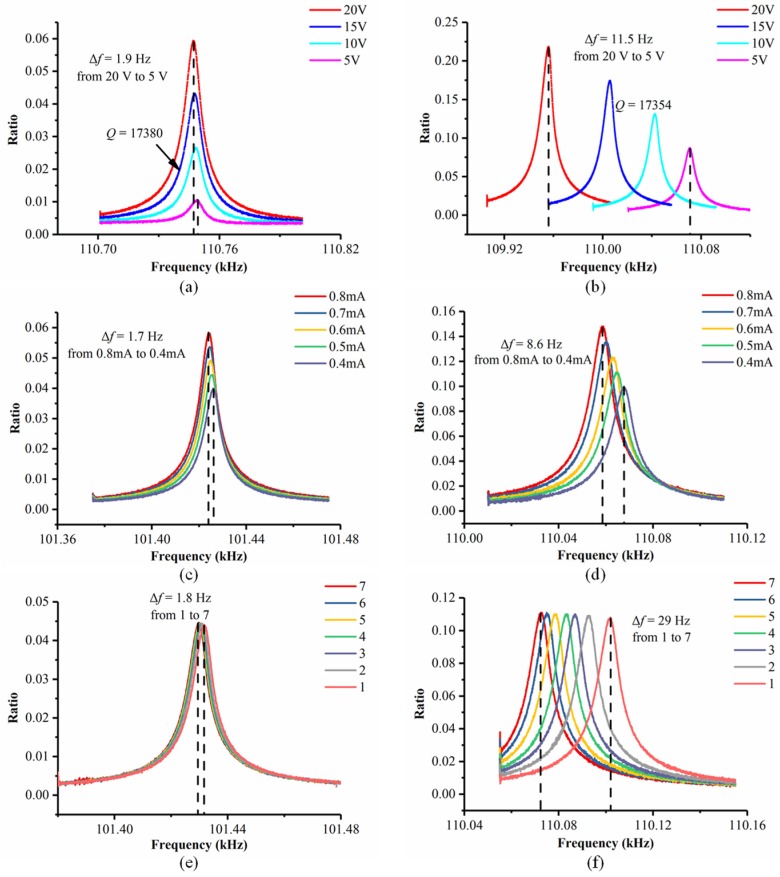
The frequency responses of resonators based on plate (**a**) and comb (**b**) drive under a group of exciting DC bias voltages. The frequency responses of resonators based on parallel-plate (**c**) and comb (**d**) drive under a group of testing currents. Multiple cycles of frequency responses of resonators based on parallel-plate (**e**) and comb (**f**) drive.

Figure 6 summarizes key results of open-loop testing where resonant frequencies of side and central resonators were measured to be ~100 kHz and ~110 kHz, respectively, with the quality factor measured as 17,380, indicating the high vacuum level of the resonant pressure sensor. Figure 6a,b show the test results of the resonant pressure sensors based on parallel-plate and comb drive, respectively, under the variation of DC bias voltage for electrostatic excitation. In comparison to the resonant pressure sensor based on parallel-plate drive, the resonant pressure sensor based on comb drive demonstrated less shifts of the resonant frequency when the DC bias voltage was increased from 5 volts to 20 volts. This is because the resonators based on comb driving showed less effects of negative stiffness compared to the parallel-plate drive resonators.

Figure 6c,d show the test results of the resonant pressure sensors based on plate and comb drive, respectively, under the variation of detecting currents for piezoresistive detection. In comparison to the resonant pressure sensor based on parallel-plate drive, the resonant pressure sensor based on comb drive demonstrated less shifts of the resonant frequency when the detecting current was increased from 0.4 to 0.8 mA. Figure 6e,f show multiple cycles of open-loop testing based on parallel-plate drive and comb drive, respectively. In comparison to the resonant pressure sensor based on parallel-plate drive, the resonant pressure sensor based on comb drive demonstrated less shifts of the resonant frequencies when the testing cycles were increased from one to seven. This is because currents can produce heat which could induce thermal stresses in the resonators, leading to the shifts of the resonant frequencies. Compared to the parallel-plate drive resonators, the resonators based on comb drive showed less frequency shifts since comb-drive electrodes possessed larger heat capacity and Euler’s critical stress.

**Figure 7:**
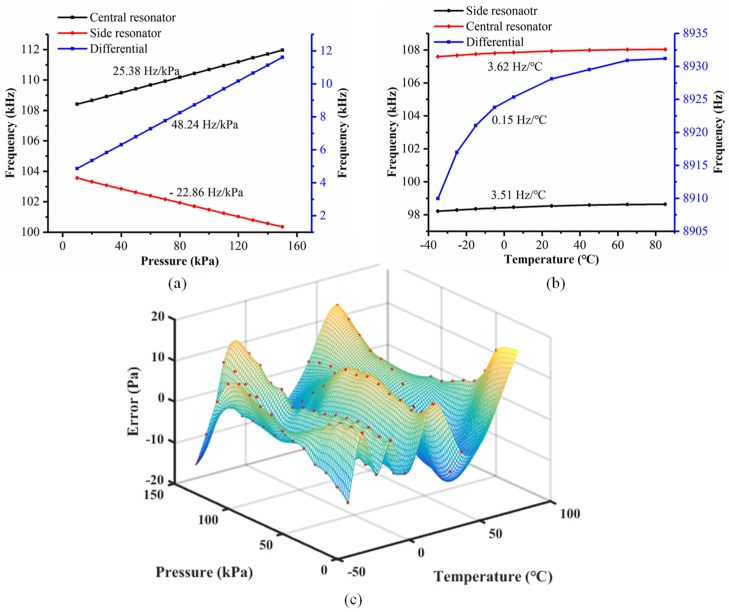
The pressure(**a**)/temperature (**b**) responses of the proposed resonant pressure sensor based on comb drive with compensated errors (**c**).

Figure 7 shows the closed-loop testing results of the resonant pressure sensor based on comb drive. Figure 7a demonstrates the resonant frequency of the sensor based on comb drive as a function of applied pressure at 25 °C. More specifically, the resonant frequencies of the central and side resonators were observed to increase/decrease with the applied pressure, showing the sensitivities of 25.38 Hz/kPa and 22.86 Hz/kPa, respectively. The differential sensitivity of the proposed pressure sensor was quantified as 48.24 Hz/kPa.

Figure 7b indicates the changes of the resonant frequencies in response to temperature variation from −35 °C to 85 °C under the application of 100 kPa atmospheric pressure, where the temperature sensitivities were quantified as 3.62 Hz/°C and 3.51 Hz/°C, for the central and side resonators, respectively, which were significantly less than the values (e.g., temperature sensitivities of 12.62 Hz/°C and 12.32 Hz/°C) reported in precious studies [13]. The temperature sensitivity with differentiation was characterized as 0.15 Hz/°C, revealing that the differential setup developed in this paper can effectively address the side effects of temperature variation to some extent.

The pressure sensor developed in this study along with the closed-loop circuit was compensated by a self-developed system under a function of pressure and temperature values, which were further adjusted using a polynomial fitting [5,15]. Figure 7c demonstrates the values of errors between the fitted and the actual pressure numbers within the pressure and temperature ranges of 10 kPa to 150 kPa and −35 °C to 85 °C, respectively. It was observed that the maximal errors and the accuracy of the resonant pressure sensor developed in this study were within ±13 Pa, and better than 0.01% F.S. (140 kPa), respectively.

## 5. Conclusions

A new resonant pressure sensor based on electrostatic excitation and piezoresistive detection was presented in this study. Two resonators driven by comb electrodes were positioned on the central and side locations of the pressure-sensitive diaphragm to eliminate the temperature disturbances on sensor performances. The proposed pressure sensor was fabricated by SOI-MEMS process. The performances of sensors were characterized by open-loop and closed-loop platforms. The frequencies of the central and side resonators were about 110 kHz and 100 kHz. The quality factor was about 17,380. The pressure sensitivities of the central and side resonators were 25.38 Hz/kPa and 22.86 Hz/kPa respectively and differential sensitivity was 48.24 Hz/kPa. The temperature sensitivities of the central and side resonators were 3.62 Hz/kPa and 3.51 Hz/kPa respectively and differential sensitivity was 0.15 Hz/kPa, which was less than resonant pressure sensor driven by parallel-plate drive electrodes reported previously. The fitting errors were better than 0.01% F.S.
